# Supplementary material for: Soluble CD26: From Suggested Biomarker for Cancer Diagnosis to Plausible Marker for Dynamic Monitoring of Immunotherapy
Source: Cancers (Basel). 2024 Jun 30;16(13):2427. doi: 10.3390/cancers16132427 (PMC11240764; doi:10.3390/cancers16132427)
Supplement: Supplementary file 1 [file cancers-16-02427-s001.zip › cancers-2980435-supplementary.pdf]

Polyclonal **rabbit anti-human CD26**

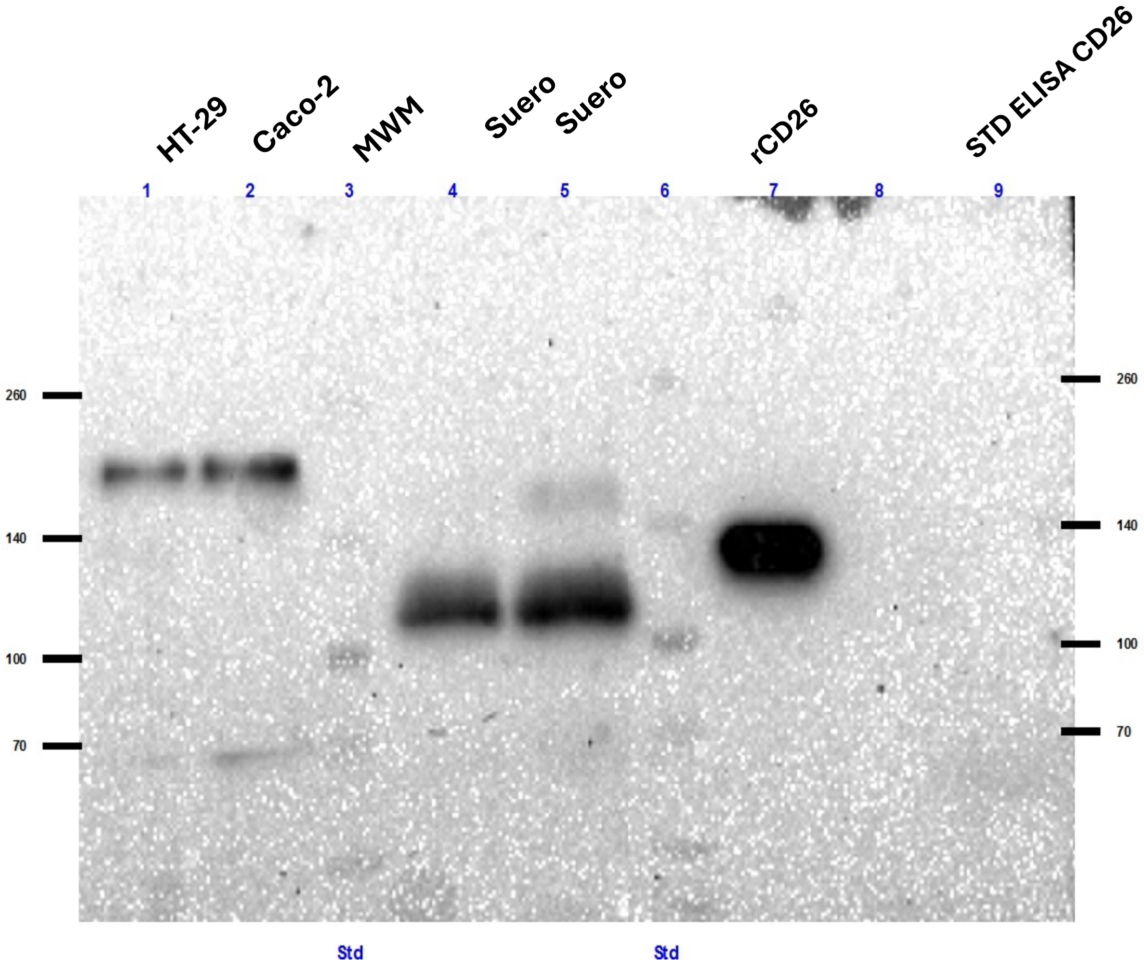

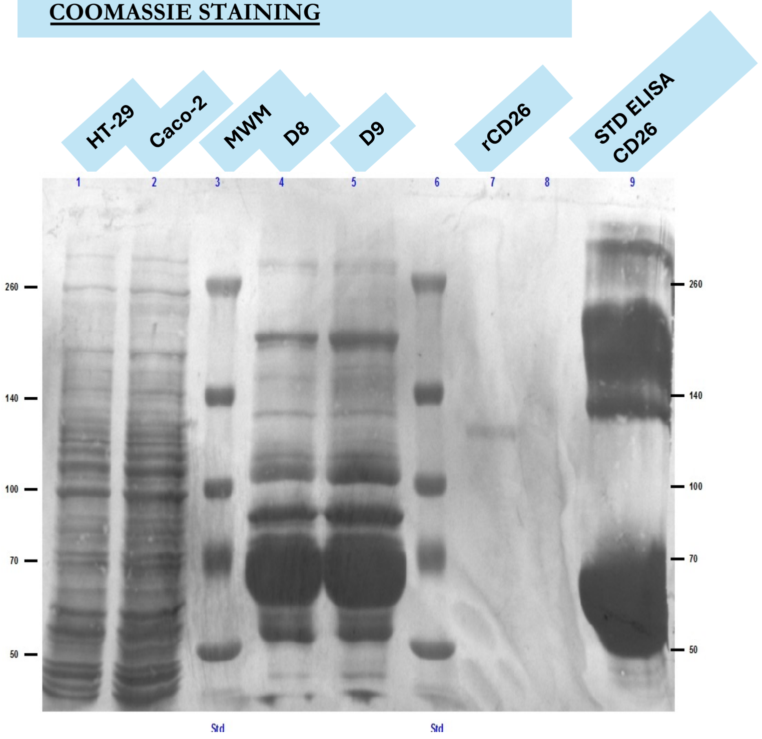

	Band No.	Mol. Wt. (KDa)
**HT-29**	1	189.77
	2	66.01
**Caco-2**	1	189.77
	2	67.34
**D8**	1	112.58
**D9**	1	168.77
**r CD26**	1	134.20

7% SDS-PAGE+WESTERN

Polyclonal goat anti-human CD26

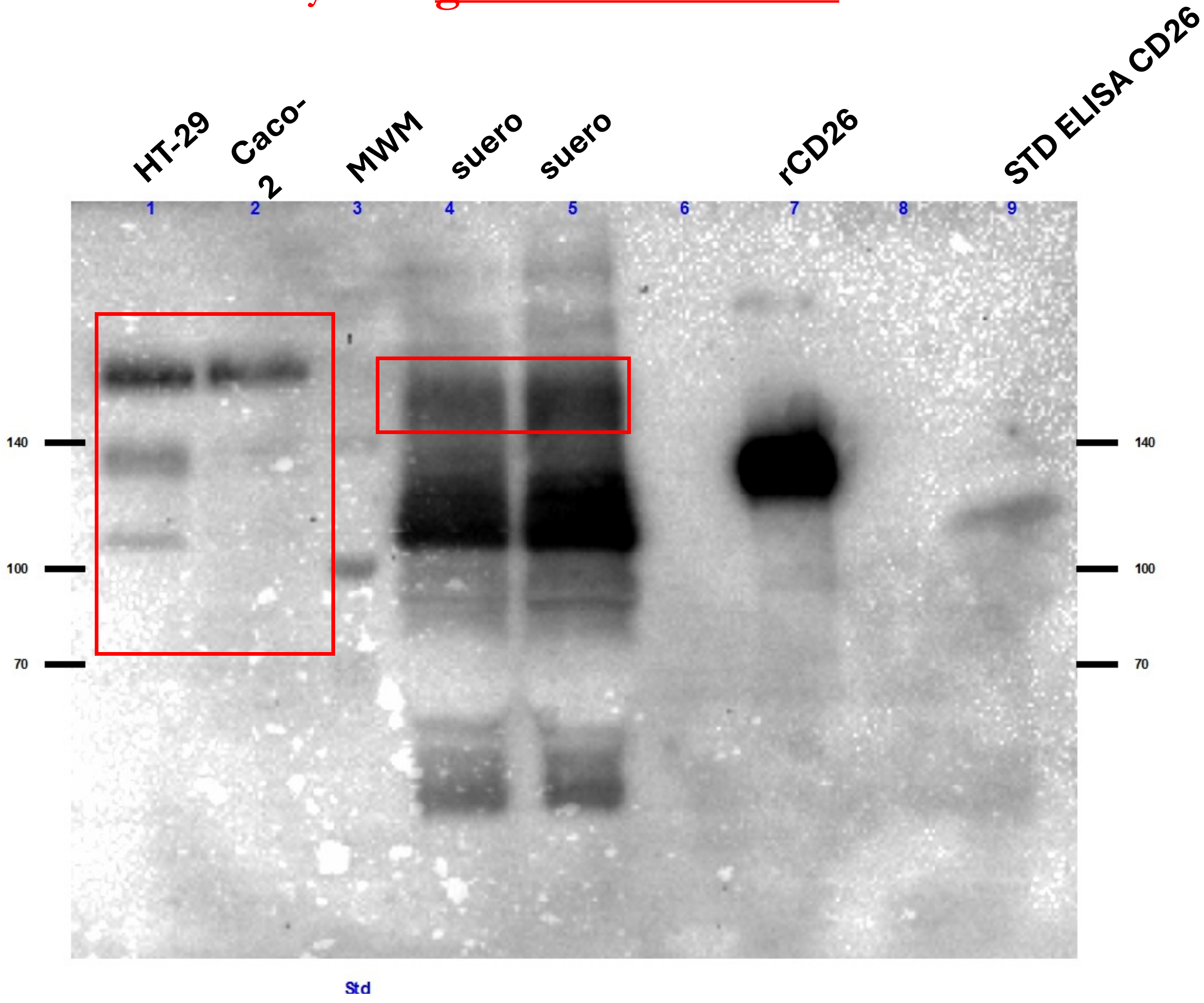

7% SDS-PAGE+WESTERN

COOMASSIE STAINING

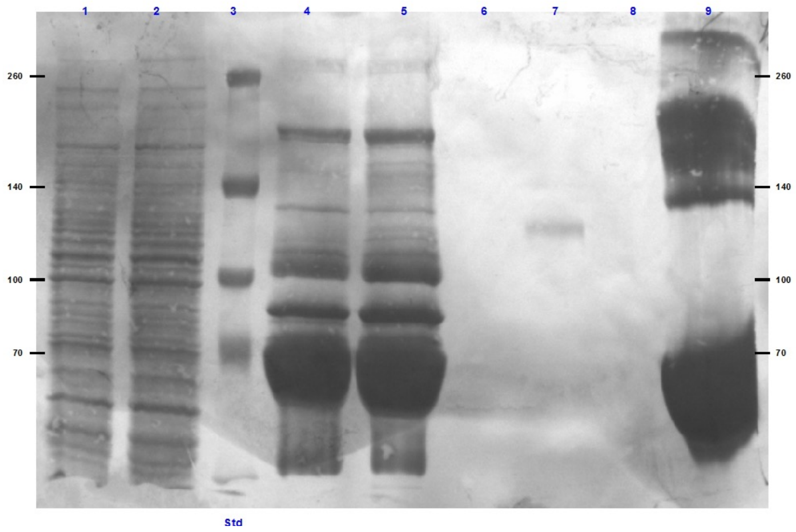

	Band No.	Mol. Wt. (KDa)
HT-29	1	174.04
	2	135.00
	3	104.73
Caco-2	1	175.89
	2	136.44
	3	106.97
D8	1	108.10
D9	1	106.97
r CD26	1	219.66
	2	132.18
STD ELISA	1	113.98

Monoclonal mouse anti-human CD26 (INMUNOSTEP)

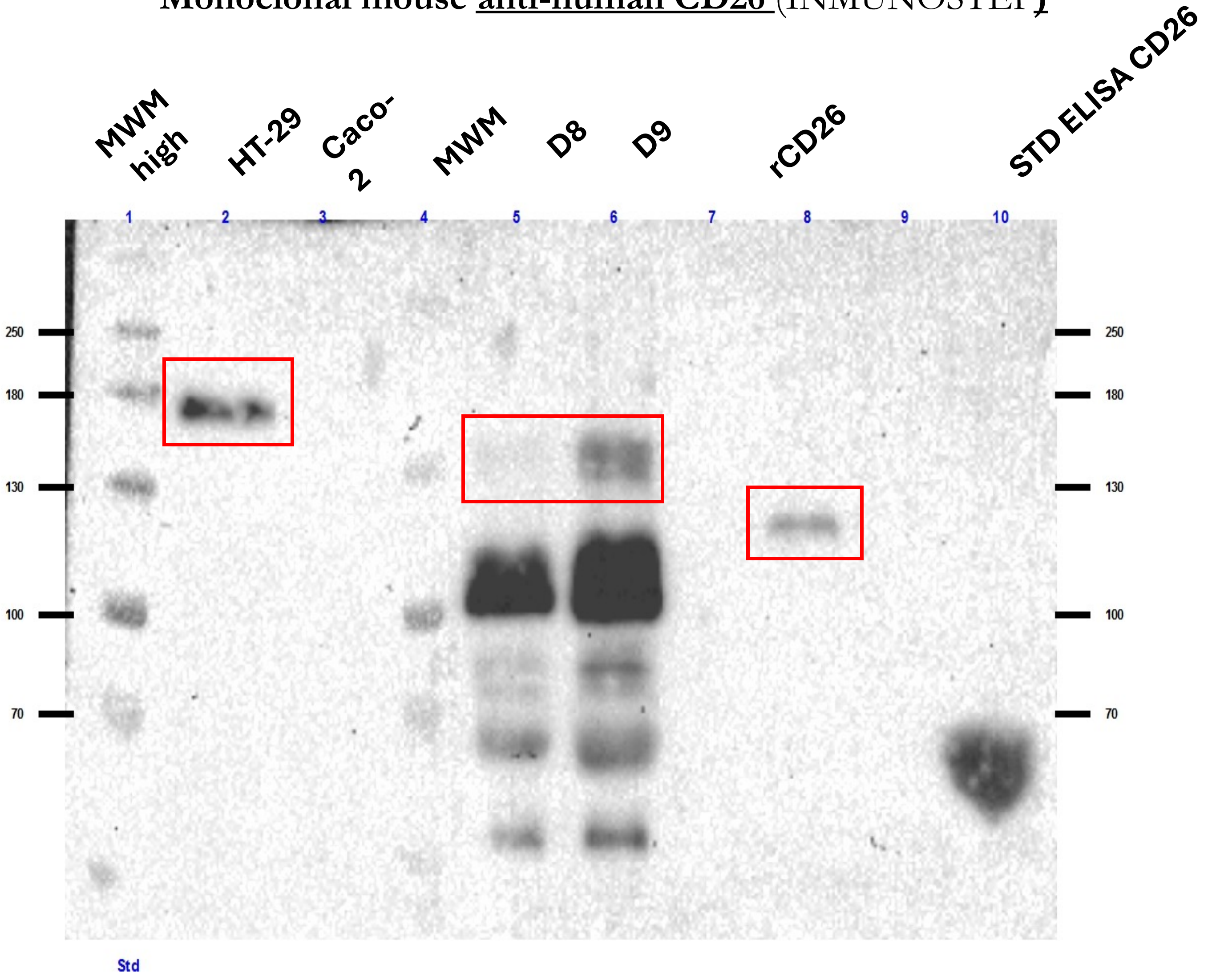

7% SDS-PAGE+WESTERN

	Band No.	Mol. Wt. (KDa)
HT-29	1	179.70
D8	1	156.19
	2	100.37
	3	63.12
	4	47.17
D9	1	157.88
	2	102.56
	3	61.24
	4	47.17
r CD26	1	125.88
STD ELISA	1	60.45
